# Biosynthesis of Nanoparticles Using Plant Extracts and Essential Oils

**DOI:** 10.3390/molecules28073060

**Published:** 2023-03-29

**Authors:** Sérgio Antunes Filho, Mayara Santana dos Santos, Otávio Augusto L. dos Santos, Bianca Pizzorno Backx, Maria-Loredana Soran, Ocsana Opriş, Ildiko Lung, Adina Stegarescu, Mohamed Bououdina

**Affiliations:** 1 NUMPEX-BIO, Universidade Federal do Rio de Janeiro, Campus Duque de Caxias Professor Geraldo Cidade, Duque de Caxias 25240-005, Brazil; 2 National Institute for Research and Development of Isotopic and Molecular Technologies, 400293 Cluj-Napoca, Romania; 3 Department of Mathematics and Sciences, College of Humanities and Sciences, Prince Sultan University, Riyadh 11586, Saudi Arabia

**Keywords:** plant extracts, polyphenols, carotenoids, essential oils, antioxidant activity, antibacterial activity, nanoparticles, green synthesis

## Abstract

Plant extracts and essential oils have a wide variety of molecules with potential application in different fields such as medicine, the food industry, and cosmetics. Furthermore, these plant derivatives are widely interested in human and animal health, including potent antitumor, antifungal, anti-inflammatory, and bactericidal activity. Given this diversity, different methodologies were needed to optimize the extraction, purification, and characterization of each class of biomolecules. In addition, these plant products can still be used in the synthesis of nanomaterials to reduce the undesirable effects of conventional synthesis routes based on hazardous/toxic chemical reagents and associate the properties of nanomaterials with those present in extracts and essential oils. Vegetable oils and extracts are chemically complex, and although they are already used in the synthesis of nanomaterials, limited studies have examined which molecules are effectively acting in the synthesis and stabilization of these nanostructures. Similarly, few studies have investigated whether the molecules coating the nanomaterials derived from these extracts and essential oils would bring benefits or somehow reduce their potential activity. This synergistic effect presents a promising field to be further explored. Thus, in this review article, we conducted a comprehensive review addressing the main groups of molecules present in plant extracts and essential oils, their extraction capacity, and available methodologies for their characterization. Moreover, we highlighted the potential of these plant products in the synthesis of different metallic nanomaterials and their antimicrobial capacity. Furthermore, we correlated the extract’s role in antimicrobial activity, considering the potential synergy between molecules from the plant product and the different metallic forms associated with nanomaterials.

## 1. Introduction

Plants and their derivatives have been used since ancient times, and have, through empirical knowledge, acquired great importance for humanity. The observations obtained from the behavior of animals that consumed plants created a kind of library in which humans began to relate the effects arising from the use of some plants [1]. Plants are called medicinal when they play an important role in curing and treating disease. In some parts of the world, these plants symbolize the only way to treat specific pathologies [2]. Historical references related to medicinal plants date from the first written documents that mention clay tablets, currently preserved in the British Museum, 3000 years before the Christian era. The well-known code of Hammurabi also described *Papaver somniferum* L. (opium), *Ferula galbaniflua Boiss and Buhse* (galbanum), *Ferula asa-foetida* L. (asafetida), and *Hyoscyamus niger* L. (henbane) [3], among many other vegetables that are still used in the treatment of diseases caused by microorganisms, such as flu [4] and bacterial infections [5]. Most molecules with healing properties present in plants are secondary metabolites involved in different processes, such as defense against pathogens and pollination [6]. Among the main substances with pharmacological action found in plants, one can highlight alkaloids, flavonoids, tannins, saponins, and terpenes [7].

Terpenes are volatile compounds, insoluble in water, and are often involved in the odors released by plants, protection, and defense against abiotic and biotic stresses [8,9]. Different pharmacological properties are associated with them, such as limonene present in citrus fruits, which has antitumor, antiparasitic, and neuroprotective activity [10,11]. The diterpene taxol was initially found in the *Taxus brevifolia* plant and is widely used in the treatment of various tumors [12], whereas menthol has analgesic, antifungal, antibacterial, and anti-inflammatory potential [13]. Alkaloids have a variety of molecules with many biological properties, such as morphine and codeine already used as analgesics; caffeine as stimulating agent; and quinones used to treat malaria [14,15]. Flavonoids are abundant and diverse and are related to numerous benefits and applications in the treatment of heart disease, inflammatory diseases, diabetes, tumors, neuronal diseases, and even the fight against aging [16,17]. Tannins are mainly associated with their astringent, antioxidant, and antimicrobial role [18,19].

The use of medicinal plants is directly related to widespread knowledge, broadly disseminated on empirical grounds and without scientific validation. This practice aims to prevent and treat diseases, use cheaper alternatives, and be less dependent on the traditional health system [20]. Despite this, such traditional knowledge of each region tends to be passed on over years of use and thus should be considered and investigated further [21]. 

There are different approaches to preparing and administering plant material, such as infusions, popularly known as teas, leaf and fruit macerates, or, more commonly, the preparation of plant extracts that can be obtained from any plant organ [22]. Each preparation methodology is indicated for extracting a specific group of molecules, which is necessary to evaluate their physicochemical parameters, such as the thermostability and solubility of the compounds to be extracted [22].

The synergy between nanotechnology and biotechnology, and bionanotechnology, emerges as an efficient alternative for various applications in a sustainable approach to the environment [23]. Nature, through the resources of medicinal plants, helps nanotechnology through biological functions that favor the interaction between the nanostructure and the medium in which it will be dispersed. Bionanotechnology presents itself as a clean and eco-friendly option, capable of solving significant difficulties. From the development of potential antimicrobial agents, with the interaction between the natural dispersive medium and the nanostructure, it operates in the food sector in search of preservatives with zero cytotoxicity and within the environmental sector with recovery techniques and environmental remediation [24,25,26].

**Figure 1:**
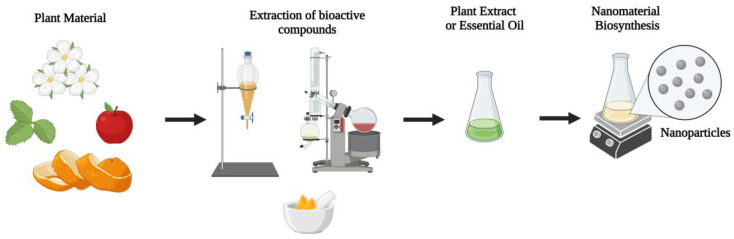
Various compounds can be extracted from different plant sources and applied to different areas, including the biosynthesis of nanomaterials. Different plant parts, such as flowers, fruits, and leaves, can be used to prepare extracts or oils. Various techniques can be employed, such as maceration and extraction using a rotary evaporator, affecting which composts will be efficiently extracted from the plant material. The plant extracts or essential oils produced can be used to synthesize nanomaterials through different protocols that may or may not involve heating.

Bionanotechnology efficiently integrates the benefits of plant extracts, such as those obtained by the aforementioned methodologies, with the high power of nanostructures. In this regard, an extract with medicinal power and high antioxidant efficiency promotes a perfect dispersive medium for stabilizing nanoparticles through its bioactivity, which simultaneously acts with its properties, thereby enhancing actions on its targets (Figure 1). This means that, for example, a plant extract has antimicrobial potential. When nanoparticles with this potential are combined, the synergistic effect between these components enhances the action and minimizes cytotoxicity and disposal impacts. This occurs because the use of precursors is reduced, as what will sustain the nanosystem is the combined actions of the nanostructure with the plant extract that they are associated with [27].

## 2. Plant Extracts

### 2.1. Polyphenols

#### 2.1.1. Polyphenol Extraction

Polyphenols represent a large group of at least 10,000 natural compounds [28] that are exclusively synthesized by plants, and that are gaining attention due to their therapeutic effects and potential technological applications. Natural polyphenols have been found in many plants and foods. Among these, important to be mentioned are fruits, vegetables, tea, medicinal plants, microalgae, edible plants, wildflowers, and cereals [29,30,31,32,33,34,35,36,37,38].

These compounds have chemical compositions related to phenolic substances, with reported bioactivities to modulate oxidative and inflammatory stress, modify macronutrient digestion, and expend prebiotic-like effects on gut microbiota. Polyphenols are almost omnipresent in plants and are generally associated with the attraction of pollinators, the implementation of structural functions, protection against ultraviolet radiation, and microbial and herbivore invasion [39,40,41]. In addition, polyphenols were demonstrated to have bioactivities and provide different benefits to human health, including cardioprotection activity, anti-inflammatory activity, anti-aging activity, kidney protective effects, activity against UV radiation, antioxidant activity, anticancer activity, antimicrobial effects, neuroprotective effects, lung protective effects, and an important role in osteoporosis [28].

The chemical structure of polyphenols is composed of at least one phenyl ring and one or more hydroxyl substituents. Phenolics’ chemical structures range from simple and small single aromatic rings to complex and heavy compact tannins [42]. The classification of polyphenols can be performed in many ways. The simplest way is a subdivision into flavonoids (the largest group of polyphenols) and non-flavonoids. They can also be subdivided into different subclasses in relation to the function of the number of phenol units within their molecular structure, substituent groups, and the linkage type between phenol units [43]. The principal groups of polyphenols are flavonoids, phenolic acids, tannins, stilbenes, and lignans [44]. Despite the wide distribution of polyphenols in plants, their properties received the attention of the scientific community only after 1995. This delay in research regarding the properties of polyphenols is because of the complexity of their remarkable variety and complex chemical structure [43].

Different methods exist for the extraction, purification, quantification, and identification of polyphenols. There is a correlation between the type of polyphenols and the protocols used for their extraction from vegetal materials [45]. There is some conventional extraction of phenolic compounds, such as heating, boiling, or refluxing. Their disadvantages are the loss of a number of polyphenols due to ionization, hydrolysis, and oxidation during the extraction process, along with the high extraction times [46]. Fundamentally, different modern methods are used for the extraction of polyphenols [45], counting pressurized liquid extraction (PLE), ultrasonic-assisted extraction (UAE), microwave-assisted extraction (MAE), supercritical fluid extraction (SFE) [47], and enzyme-assisted extraction (EAE) [48]. Equivalent to that in modern methods, conventional liquid–liquid and solid–liquid extraction [47] and high hydrostatic pressure (HHP) extraction procedures are often used [49]. One of the latest techniques developed for polyphenol extraction is pulsed electric fields (PEF) [50].

Experimental parameters such as time, temperature, choice of organic solvents, solvent-to-feed ratio, and the number of repeated extractions of the sample play a significant role in the extraction yield. The water solubility of polyphenols can be reduced because they are attached to the cell wall matrix through a glycosidic/ester linkage. Solubility is strongly influenced by extraction time and temperature. A higher temperature increases the solubility and mass transfer rate the compounds of interest. At the same time, the viscosity decreases, and the surface tension of the solvents enables a higher extraction percentage [51,52,53].

Thus, several studies demonstrated that an antioxidant is needed as a basic stabilizer for extracting polyphenols [51]. For example, methanol and ethanol are the most used solvents to extract polyphenols [47]. In addition, it is essential to select a solvent with low viscosity for accelerating mass transfer [45]. To avoid the extraction of unwanted compounds such as chlorophyll, waxes, fats, terpenes, sugars, organic acids, and proteins, extra extraction steps must be introduced to the extraction protocol (for example, solid phase extraction, SPE) [54,55].

Polyphenolic compounds can be extracted from plant samples that are fresh, frozen, or air-dried. Before the extraction, the vegetal material needs to be ground, dried, and homogenized. The selection of freeze-drying of the plant samples enabled a higher concentration of the phenolic compound than the selection of air drying, which directly impacts their content [56].

##### Conventional Techniques Used for the Extraction of Polyphenols

Even if conventional polyphenol extraction methods have several disadvantages, they are still the most commonly used due to their ease of use, efficiency, and broad range of applicability [57,58]. In general, liquid–liquid extraction is used to separate phenolic compounds from industrial liquid by-products, such as those from the beverage industry [51].

In the case of conventional methods for the extraction of polyphenols, alcohols (methanol, ethanol), acetone, diethyl ether, and ethyl acetate are frequently mixed with water. Among the disadvantages of the conventional methods are the possible hazardous effects of the solvents used on human health, the environment, and the remaining solvent residues in the final extract, which includes additional extraction steps, time, and costs. Furthermore, polyphenols could not be completely extracted by polar organic solvents with respect to phenolic acids (benzoic, cinnamic acids). Thus, in such cases, it is necessary to use water–alcohol mixtures. It is also important to notice that nonpolar compounds (for example, waxes, chlorophyll, sterols) may be extracted by polar solvents (for example, benzene, hexane, chloromethane, chloroform) [57,58]. It was demonstrated that methanol was more efficient for extracting polyphenols with lower molecular weight, and acetone was suitable for extracting flavanols with a higher molecular weight [59,60,61,62].

Conventional extraction methods used for polyphenols are usually carried out at a temperature ranging from 20 to 50 °C; high temperatures need to be avoided because of their rapid degradation (for example, anthocyanins). Furthermore, long extraction times should be avoided for the same reason. Other, more popular conventional methods are maceration and Soxhlet extraction. However, their disadvantages are their low efficiency use of large volumes of organic solvents, and that they are hazardous to the environment [47].

##### Modern Techniques Used for the Extraction of Polyphenols

Ultrasonic-Assisted Extraction (UAE) is a novel extraction technique compared to conventional ones. This technique is easy to handle and requires a moderate volume of solvent, and thus is very economical [63,64]. The UAE technique uses high-power and low-frequency sound waves to detach the solute from the vegetable matrix. The sound waves that pass into the solvent extraction conduct some high/low-pressure cycles, further producing cavitational bubbles. The bankruptcy of the cavitational bubbles generates energy, leading to a higher infiltration of the extraction solvent into the cellular vegetal material and better mass transfer to and from interfaces [65]. Thus, UAE has fewer instrumental demands [66] and a short extraction time, obtains high yields, and is environmentally friendly [67].

Another alternative modern technique is the microwave-assisted extraction (MAE) technique, which analytical chemists frequently use to isolate and extract phytocompounds. MAE has multiple advantages, such as the use of small amounts of solvent extraction and minimum extraction times; as well, it can process a large number of samples simultaneously. Microwaves are non-ionizing radiation, with frequencies ranging between 300 and 300,000 MHz, capable of stimulating the molecules’ rotational energy levels. In general, for scientific and domestic purposes, microwave frequency is 2450 MHz [68]. Two facts characterize MAE: ionic conduction and dipole rotation, which occur, in most cases, in parallel in polar materials and solvents [69,70]. This results in an increased solvent temperature and a rising solute solubility [71]. Compared to other traditional extraction techniques, the use of MAE showed excellent performance in terms of recoveries and precision [68].

Supercritical fluid technologies have been extensively used due to the selective isolation of antioxidant compounds from vegetal material and mild conditions that can avoid the oxidation/degradation of the sensitive compounds [72,73]. Considering that supercritical fluid solvents are intermediate between liquids and gases, an increased solubility is usually reached with the fluid’s increasing density. The most important advantage of this extraction technique is the possibility of changing the characteristics of the solvent close to its critical point [52].

Small variations in pressure rapidly change solvent properties [74]. The advantages of this extraction method are that it is an environmentally beneficial alternative to conventional organic solvent extraction, it is rapid, automatable, and selective, and it avoids high volumes of toxic solvents. In addition, the absence of air and light during the procedure lessens the degradation processes that can appear during traditional extraction techniques [75]. This extraction technique also replaces organic solvents such as n-hexane, dichloromethane, chloroform, and others that are conventionally used. Until now, the most used critical fluid has been supercritical carbon (SC–CO_2_) because it is environmentally friendly, non-flammable, compatible with food products, can be easily separated from solutes, and is a low-cost technique [51]. Bleve et al. demonstrated that the extraction of anthocyanins using SC–CO_2_ methods required high pressure and the presence of an organic co-solvent in high percentages due to the polarity of anthocyanins [76].

The PEF (pulsed electric field) technique electroporates cell membranes, obtaining a high extraction yield. In the case of PEF, a minimal amount of energy is used. This is a non-thermal technique and is also an environmentally friendly extraction technique. Thus, many thermally unstable polyphenols are maintained completely in the resulting extracts. PEF uses moderate to high electric field strength, ranging from 100–300 V/cm in batch mode and 20–80 kV/cm in continuous mode extraction [75]. It is also important to mention that this extraction technique is comparable regarding its efficiency to other modern techniques, such as UAE [50]. Various modern polyphenol extraction techniques are presented in Table 1.

#### 2.1.2. Techniques Used for Polyphenol Analysis

Many spectrophotometric methods were developed to quantify the phenolic compounds from vegetal materials. Spectrophotometric methods offer important qualitative and quantitative information. Thus, a well-known classical method for the determination of total phenolic and flavonoid content is the method which uses the Folin–Ciocalteu reagent and aluminum chloride complexation [85]. Through this method of determination, the Folin–Ciocalteu reagent interacts with other reducing non-phenolic compounds, conducting a supra evaluation of the total phenolic compound present in the vegetal material sample [86,87]. The main disadvantage of spectrophotometric determination is that it only estimates the total phenolic content. This determination technique does not separate or give a quantitative measurement of the individual compounds [51].

High-performance liquid chromatography (HPLC) and gas chromatography (GC) are modern techniques for the qualitative and quantitative determination of phenolic compounds in various matrices [87]. The coupling of the HPLC system with ultraviolet (UV) detection, electrochemical detection, mass spectrometry (MS), or electron ionization mass spectrometry is the most frequently used method for determining phenolic compounds. In addition, GC coupled with MS, high-speed counter-current chromatography, chiral capillary electrophoresis, or Fourier transform near-infrared reflectance spectroscopy is used for the same purpose. However, HPLC determination of polyphenols has a limitation mainly in a complex matrix such as that of plant extracts. In this case, before analysis, preconcentration and purification steps using SPE cartridges are needed. Polyphenols are generally purified using an adsorption–adsorption process using C18 and copolymeric extraction cartridges [51].

The use of the methods with the previously mentioned coupled systems offers information regarding the molecular mass and structural characteristics of the compounds of interest. Because the phenolic compounds present low volatility, their GC analysis needs a previous derivatization step [88]. Generally, chromatographic methods are preferred to other techniques for separating and quantifying polyphenols, even though they are expensive to buy and maintain. The HPLC coupled with the UV detector was found to be more convenient to run for routine analysis [89]. Some HPLC experimental conditions used to determine of some classes of phenolic compounds are presented in Table 2.

### 2.2. Carotenoids

#### 2.2.1. Carotenoid Extraction

Carotenoids (also called *tetraterpenoids*, a term derived from their chemical composition—8 isoprene molecules and 40 carbon atoms) are a class of brightly colored natural pigments (yellow, orange, or red) produced by plants and algae, as well as by several bacteria and fungi [95]. The scientific community identified more than 700 different carotenoids derived from the C40 isoprenoid skeleton that can be further categorized into two classes: carotenes (purely hydrocarbons, without oxygen in structure) and xanthophylls (containing oxygen in structure). The carotenoids give the characteristic color to pumpkins, carrots, parsnips, corn, tomatoes, canaries, flamingos, salmon, lobster, and shrimp and are a group of phytochemicals important for human health. However, despite a large number of carotenoids present in nature, only a few are in the human diet, and some epidemiological and clinical studies suggest that carotenoid consumption is associated with lower risk in the case of aging, including a lower risk of cancer, cardiovascular disease, cataracts, and age-related macular degeneration or eye disease [96].

Carotenoids have been intensively studied due to their role in photobiology, photochemistry, and photo medicine, and their usage, as colorants and antioxidants, in the food industry (manufactured foods, drinks, and animal feeds). They are used as natural extracts (for example, as tomato, paprika, or marigold extracts) and as pure compounds obtained by chemical synthesis. It is important to note that carotenoids are believed to play an important role in plant physiology and are involved in photosynthesis [97,98].

These compounds are frequently described as provitamin A, because vitamin A is obtained after the carotenoid metabolism. However, although more than 700 carotenoids have been identified in the last decade, only 50 possess provitamin A activity, and less than 40 are present in the human diet. From this, only 14 along with some of their isomers have been identified in blood and tissues [99].

Mixed carotenoids (including *α*- and *β*-carotene) can be isolated from algae, *β*-carotene from palm oil, and lutein and lutein esters from marigolds (the most predominant in leafy vegetables, but the color is masked by its co-existence with chlorophyll); lycopene is a characteristic of the tomato’s composition. At the same time, fruits contain varying proportions of cryptoxanthin, lutein, and antheraxanthin [100].

*β*-carotene has already been synthesized for many years, while lycopene has only been synthesized in recent years. The commercial synthetic formula of carotenoid supplements contains only *β*-carotene in the form of all-trans isomers. In contrast, *β*-carotene from a natural source, such as algae, fruits, and vegetables, is composed of all-trans, 9-cis, and other cis-isomers [101].

The literature data show dehydrated peppers are the richest source of *β*-carotene, followed by carrots and grape leaves. The fresh leaves of spinach, sweet potato, dandelion greens, and turnip greens are rich in lutein and zeaxanthin, while tomato is the most abundant source of lycopene [102]. We can conclude that carotenoids have two important roles in plants and algae bio life: they absorb blue light that will be used in photosynthesis and protect the chlorophyll from photodamage. In human life, they act as vitamin A (especially *β*-carotene, *α*-carotene, and *β*-cryptoxanthin) and as antioxidants [103].

Analytical methods are an essential factor for accurately estimating carotenoid contents due to the ease of degradation of this compound in the presence of solvents. For this, it is important to establish the correct solvent use for extractions according to the polarity range between carotenoid and solvent. Therefore, researchers have established that xanthophylls are better extracted using polar solvents (for example, alcohols and acetones), whereas carotenes are extracted by nonpolar solvents (including edible fats and oils) [104,105].

For this reason, in some cases, the carotenoid content is often underestimated depending on the nature of the solvent used and the extraction procedure used [106].

The literature describes many extraction methods to obtain carotenoids. Extraction is based on cell disintegration and occurs at different temperatures and pressures, as follows:

*1.*  *Room temperature and atmospheric pressure*: maceration process;
*2.*  *Boiling temperature of the solvents and atmospheric pressure*: extraction of carotenoids using a Soxhlet technique in the presence of the proper solvent;
*3.*  *Low temperature and high pressure*: accelerated solvent extraction (ASE), also known as pressurized liquid extraction (PLE); supercritical fluid extraction (SFE)—occurs with minimal use of a co-solvent, such as ethanol;
*4.*  *Ultrasound and microwaves, high voltage pulses that facilitate the release of intracellular carotenoids*: microwave-assisted extraction; ultrasound-assisted extraction (UAE); pulsed electric field-assisted extraction (PEF); enzyme-assisted extraction (EAE).

Some authors have advanced the hypothesis that maceration is superior to other methods, probably due to the minimization of carotenoid degradation working at room temperature. On the other hand, the Soxhlet method is a conventional technique providing the highest recovery of carotenoids and presents good potential for obtaining a promising reaction efficiency due to the low viscosity and surface tension at boiling temperature, which improves the solubilization of carotenoids in the used solvent. At the same time, this has the disadvantage of using significant amounts of solvents and a long process time, increasing the extraction cost [104,107,108].

The SFE technique uses CO_2_ as solvent and ethanol as co-solvent, which can be classified as a “green technique”. It is a non-thermal method, efficient, and selective for carotenoids, especially for xanthophylls (polar carotenoids). UAE, pressurized liquid extraction, PEF, and EAE are also non-thermal and rapid methods for the extraction of carotenoids, but it has not yet been established if they are less costly, environmentally friendly, efficient, or reproducible. Therefore, these methods need to be further evaluated [109,110,111].

#### 2.2.2. Carotenoid Analysis

Chemical (specific color reactions) and spectral methods can be used to identify the obtained carotenoids. High-performance liquid chromatography (HPLC) is usually used to determine carotenoid components, presenting the advantages of using low quantities of substances and high separation speeds and the disadvantage of cost. Spectral methods are preferred due to their high sensitivity, perfect result reproducibility, sample integrity conservation, and needing less time. Visible spectroscopy (UV–VIS), IR spectra, RAMAN spectra, and mass spectrometry (MS) are the spectral methods most used to detect carotenoids. Along with these methods, microwave spectra (which give information on the repartition of electronic density in the molecules of carotenoids, allowing the evaluation of the hybridization degree, but which have a limit because they can be used only in the case of molecules with permanent electric moment) and nuclear magnetic resonance spectra (which determine the structure of carotenoids) are also sometimes used [112].

### 2.3. Essential Oils

#### 2.3.1. Essential Oil Extraction

Essential oils are secondary metabolites isolated from different parts of plants, such as leaves, flowers, peels, buds, seeds, stems, roots, barks, and pods [113,114]. They are a complex mixture of polar and nonpolar compounds, containing from a few dozen to several hundred constituents. Chemically, essential oils consist of hydrocarbons (terpenes and sesquiterpenes) and oxygenated aromatic compounds (alcohols, aldehydes, ketones, acids, phenols, oxides, lactones, acetals, ethers, and esters) [115,116,117]. They are used in pharmaceuticals, perfumes, cosmetics, food, drink flavoring, cleaning products, and pesticides [118,119]. Essential oils obtained from medicinal plants have health benefits, being used as anti-cancer agents, anti-viral agents, and antibacterial agents, and for their anti-diabetic effects, antifungal effects, anti-spasmodic effects, and anti-inflammatory and antioxidant activity [115,120].

The composition of essential oils from plants is affected by the part of the plant analyzed, growing conditions, variation of species, plant growth conditions, extraction methods, place of origin, and phase of plant growth [116,121]. Among these, the extraction method is an important component in improving the overall yield and quality of the essential oil, which should be low-cost and efficient [122].

##### Conventional Extraction Methods

Conventional extraction methods for essential oils are characterized by high energy consumption, production of some aerosols and greenhouse gases, and long extraction time [120]. Although they have several disadvantages, conventional extraction methods are still used today.

*Maceration*

In this method, oils are used as solvents to extract the essential oils from plant material. The extraction by maceration occurs in a closed vessel by putting the oil in contact with the cut and crushed plant material. The mixture is stirred occasionally, and the liquid is separated after a week. It can be kept in an airtight container in a cool and dry place for up to 12 months. It was observed that the essential oil obtained by maceration is more beneficial than the one obtained by distillation because it captures heavier and larger plant molecules than those captured in the distillation process [123].

*Enfleurage*

Enfleurage is the oldest extraction method for essential oils from flower petals, implementing the use of fat. Vegetable or animal fats must be odorless and solid at room temperature [124]. During this method, a cold fat is spread on the flowers, which will absorb the odors emanating from them. The process is repeated until the fat is saturated, replacing the old flowers, after exhaustion, with new ones. After that, the fat is removed and the essential oil is extracted with alcohol, obtaining pure oil after the alcohol evaporates. In hot enfleurage, the difference is that fats are heated to 40–60 °C [125].

*Cold-press extraction*

Expression, or cold pressing, is the oldest extraction method used to extract essential oils from plants, flowers, seeds, and citrus [122,126]. This method is used to extract the citrus peel’s essential oils due to the aldehydes’ thermal instability [119]. The expeller-pressed oil, at low temperature and pressure, is 100% pure and retains all plant characteristics [122,127]. First, the whole fruit is placed in a device and then mechanically pressed when the essential oil glands are crushed or broken to release the oil. As a result, this process produces an aqueous emulsion, which is later centrifuged to separate the essential oil that rises to the surface [127].

*Steam distillation method*

Steam distillation is the most commonly used method for extracting essential oils from plants [128]. The principle of the method consists of distilling the mixture of compounds at a temperature lower than their boiling point. The extraction time can vary between 1 and 10 h [119]. The dried and chopped plants are placed in a bed above the steam source on a perforated grid. Under the heat, the structures of the plant materials degrade and rupture, releasing the essential oils through evaporation [129]. Steam and vapors from the oil are condensed and separated in a vessel called a florentine flask [119,120]. Steam distillation is a straightforward process, but it has many drawbacks, among which are the production of greenhouse gases, the long extraction time, and the possibility of the formation of artifacts due to the high temperature or the acidity of water [119,120]. In order to meet energy demands and reduce pollution, studies have been conducted on steam distillation technologies. Thus, a hybrid solar steam distillation system [130,131,132], an ohmic accelerated steam distillation method [133], and a microwave-accelerated steam distillation method [134] were used for essential oil extraction.

*Hydro-distillation*

Hydro-distillation, discovered by Avicenna, is the oldest and simplest method for extracting essential oils from dried plant material such as wood or flowers. The process involves immersing the plant material in water and then boiling the entire mixture, the extraction principle being based on isotropic distillation. As the water boils, the mixture must be stirred, thus avoiding clumps of material that would otherwise settle to the bottom and thermally degrade. Water and essential oils evaporate and are condensed into an aqueous fraction, separating them in a decanter [135]. Among the advantages of this technique are the prevention of overheating of the plant material and the ability to isolate plant materials below 100 °C [120,136]. However, the method is somewhat slow, with a risk of thermal degradation, and requires energy for heating. According to the new technologies, the modified hydro-distillation process (using microwave-assisted hydro-distillation) [137,138,139,140,141] and ohmic-assisted hydro-distillation were used to extract essential oils [137,142].

*Hydro-diffusion*

Hydro-diffusion extraction differs from steam distillation by supplying steam from the top of the generator and not from the bottom, as in the case of steam distillation. In addition, in this process, only dry plants are used, which are not damaged at the boiling temperature [143]. The steam passes through the plant material on a grid, and the mixture of water vapor and oil condenses on coils fixed below it. This mixture is separated in the same way as in steam distillation. The process can be carried out at low pressure or in a vacuum when the steam temperature can be reduced below 100 °C [136,143]. This approach reduces the extraction time, increasing the yield simultaneously [144]. An innovative microwave hydro-diffusion and gravity method [145,146] and microwave steam diffusion were recently used to extract essential oils [147].

*Solvent extraction method*

This process is used to extract heat-sensitive essential oils, using petroleum, acetone, ether, methanol, ethanol, and hexane as solvents [128,136]. The solvent is mixed with the plant material and then heated to extract the essential oil. After extraction, the liquid mixture containing the essential oil is filtered. The filtrate, which is a combination of wax, fragrance, and essential oil, is then mixed with pure alcohol to dissolve the essential oil in it. After distilling the alcohol at a low temperature, absolute oil remains. Although the method is relatively simple and uses a lower temperature, thus reducing the risk of chemical changes, it presents disadvantages, such as the long extraction time, being more expensive, requiring a relatively high consumption of solvents, and often unsatisfactory reproducibility [148,149].

##### Modern Extraction Methods

The long extraction time, lower efficiency, high solvent requirement, high energy consumption, and other disadvantages of conventional methods, and the need to reduce carbon dioxide emissions, led to the development of non-conventional methods. These require a shorter extraction time, lower costs, and produce a higher yield [150,151].

*Solvent-free microwave extraction*

The losses of several evaporative constituents and toxic solvent residues at the final product stage of ordinary extraction techniques led to the development of a solvent-free microwave extraction method. This method, developed by Cheat and co-workers, consists of extracting essential oils without adding solvent [127,152,153]. It consists of dry plant distillation at atmospheric pressure without water or any organic solvent [154]. This method’s advantages are high selectivity, short extraction time, higher extraction rates, and many others [146]. 

*Supercritical fluid extraction*

Supercritical fluid extraction is a relatively new technique for obtaining essential oils, using supercritical fluids as the extraction solvent. The most commonly used supercritical fluid is CO_2_ because it is non-flammable, non-explosive, non-toxic, accessible at low cost, high purity, easily removed from extracts, and diffusivity is two or three times higher than other fluids [122,136]. The heated and compressed fluid passes through the plant material to extract the compounds from the plant, after which the mixture of carbon dioxide and plant extracts is sent to two separators to separate the plant extracts from the CO_2_ by decompression. Therefore, this method obtained essential oils without thermal or hydrolytic damage [125].

*Subcritical water extraction*

Subcritical water extraction, also called pressurized hot water extraction or pressurized low-polarity water extraction, uses water in the subcritical state at temperatures between 100–374 °C and pressure high enough to maintain the liquid state [155]. For the first time, this method was used by Basile et al. [156] for the extraction of volatile oils from rosemary leaves. This method’s advantages include simplicity, cost-efficiency, short extraction time, high quality of the extract, and environmental friendliness [157,158].

*Ultrasound-Assisted Extraction*

Ultrasound-assisted extraction extracts volatile oils from flowers, leaves, or seeds [127]. Ultrasounds have mechanical vibrations, which, as they move, create expansion and compression cycles within the medium [159]. A cavitation phenomenon is created by microscopic bubbles’ production and breakdown, leading to cell membrane damage [122,160]. Ultrasound-assisted extraction can occur in an extraction bath or a glass reactor with a conical ultrasonic system. The method involves subjecting plant material immersed in water or solvent to ultrasound. This method’s advantages include reducing the extraction time, being more efficient, increasing the extraction yield, and requiring less energy consumption [161].

#### 2.3.2. Essential Oils Evaluation

The most used methods for analyzing essential oils are chromatographic (thin layer chromatography and gas chromatography) and spectroscopic (Fourier transform infrared spectroscopy).

*Thin layer chromatography*

Thin layer chromatography (TLC) is a simple, fast, and inexpensive procedure that can analyze very complex mixtures, both qualitatively and quantitatively. Determinations of volatile oils by thin layer chromatography have been carried out over time on silica gel plates. Different systems can be used as mobile phase, such as acetone–hexane (1:30), hexane–dichloromethane (1:1.3) [162], toluene–ethyl acetate (93:7, *v*/*v*) [163], petroleum ether–cyclohexane–ethyl acetate–acetone–methanol (6:1.6:1:1:0.4) [164], benzene–glacial acetic acid (1:1), and carbon tetrachloride–acetone–glacial acetic acid (15.2:3:1) [165]. The spots can be visualized by spraying the plate with anisaldehyde–sulfuric acid solution, with phosphomolybdic acid or vanillin/sulfuric acid [166].

*Gas chromatography*

Essential oils, due to their volatile nature, require sophisticated analysis techniques, of which gas chromatography is the most used for quantitative and qualitative analysis. Gas chromatography coupled with mass spectrometry (GC-MS) and flame ionization detection (GC-FID) are most often used to analyze essential oils. These two techniques (GC-MS and GC-FID) combine the characteristics of gas chromatography and those of mass spectrometry, respectively, with flame ionization detection to identify the compounds from the sample [123]. These techniques reduce the possibility of error, and the identification of substances is more precise.

*Fourier transform infrared spectroscopy (FTIR)*

FTIR, used for both qualitative and quantitative purposes, is an alternative to classical methods based on gas chromatography. The spectra for plant extracts are very complex because they are mixtures of several components, and the assignment of bands can be difficult. This method requires minimal sample preparation, and IR spectrometers are easy to use and relatively inexpensive, allowing a rapid, non-destructive, and cost-effective evaluation of essential oils [167]. Attenuated total reflectance-Fourier transform infrared (ATR-FTIR) spectroscopy is preferred over FTIR spectroscopy because it requires no sample preparation and, being placed on a high-refractive-index crystal cell, can be easily recovered [168]. In ATR, the beam is reflected to the samples several times, making it possible to examine poorly absorbing samples [169].

### 2.4. Antioxidant Activity

The new tendency in synthesizing nanomaterials is to decrease the quantities of chemicals used that could affect the environment, decreasing the time and energy consumption for preparing nanomaterials with controlled properties (for example, surface area, structure, activity, and shape). With this aim appeared the green synthesis of metal and metal oxide nanoparticles, trying to minimize the negative effects of chemicals using plant extracts instead of different substances. Strong bases such as sodium borohydride and sodium hydroxide and capping agents or stabilizers such as cetyltrimethylammonium bromide are frequently used for nanoparticle preparation. These can be changed by extracts from different parts of plants with antioxidant (reduction) properties that transform the metal salts into metal oxides or metal nanoparticles. The most used compounds in green synthesis are polyphenols, citric acid, vitamins (B, C, D, K), citric acid, and enzymes. Furthermore, their role is to prevent the aggregation of nanoparticles, being active components in the growing and stabilization stages that follow the degradation of metal salts [170]. Antioxidants are substances that delay or inhibit the oxidation reaction (producing free radicals). The total antioxidant capacity (TAC) assays by different methods can be classified by mechanism and methods used for evaluation.

#### 2.4.1. Methods for TAC Assessment According to Mechanism

The mechanism of TAC evaluation can be hydrogen atom transfer (HAT), electron transfer (ET), or, in some cases, these two mechanisms may not be clearly differentiated.

*HAT-based methods*—consist of the capability of an antioxidant to neutralize free radicals by proton donation. Usually, phenols (ArOH) transfer a proton to peroxyl radicals, as follows [171]:

ROO^•^ + ArOH → ROOH + ArO^•^ (1)

Some methods that are based on the HAT mechanism include ORAC (oxygen radical absorbance capacity), TRAP (total radical-trapping antioxidant parameter), and Total Oxidant Scavenging Capacity (TOSC) [171]

*ET-based methods*. This mechanism depends on solvent and pH, and it is relatively slower than reactions based on HAT. The reaction for ET is based on [171,172]:

ROO^•^ + ArOH → ROO^−^ + ArOH^•+^ (2)

ArOH^•+^ + H_2_O ↔ ArO^•^ + H_3_O^+^ (3)

ROO^−^ + H_3_O^+^ ↔ ROOH + H_2_O (4)

Some methods that use the ET mechanism are Ferric Reducing Antioxidant Power (FRAP) and Copper Reduction Assay (CUPRAC, AOP-90) [172].

*HAT and ET-based methods.* It is possible for radicals to be neutralized by direct reduction via electron transfers or by radical quenching via H atom transfer. TEAC (Trolox equivalent antioxidant capacity) or other ABTS assays and DPPH (2,2-diphenyl-1-picrylhydrazyl-hydrate) are methods based on both mechanisms. TEAC and ABTS assays use the 2,2’-azinobis-(3-ethylbenzothiazoline-6-sulfonic acid) (ABTS^•+^) radical. Trolox (6-hydroxy-2,5,7,8-tetramethylchroman-2-carboxylic acid) is a water-soluble analog of vitamin E, used as a standard antioxidant for all these methods [172].

#### 2.4.2. Methods Used for Evaluation of TAC

*Spectrometry*

This category includes methods such as DPPH, ABTS, ORAC, CUPRAC, and TRAP, which are based on the reaction of antioxidants with a radical, followed by the determination of end products by colorimetry, fluorescence, and chemiluminescence measurements [173].

*Electrochemistry*

This category includes cyclic voltammetry, amperometry, and biamperometry [173].

*Chromatography*

Gas chromatography and high-performance liquid chromatography are mostly used. These techniques separate the compounds with antioxidant properties, followed by a reaction with radicals and detection of the resulting compound or its spectrophotometric or fluorescence assessment [173]. A frequently used method for TAC assessment is spectrophotometric because it is a rapid and low-cost method and the analytical instruments necessary are the simplest in comparison to other methods. The photometric methods are followed by voltammetry, which offers low detection limits, fast analysis, and simple sample preparation [173].

### 2.5. Antibacterial Activity

The urgency of developing new drugs and antimicrobial technologies is due to the increase in cases of antibiotic resistance, and studies suggest that there will be ten million deaths per year by 2050, on average, due to infections caused by these resistant microorganisms [174]. Bacterial infections can affect different organs and tissues. In addition, these pathogens can acquire resistance through different mechanisms, such as efflux pumps, the formation of bacterial biofilms, and enzymatic activation of antibiotics, among other mechanisms [175]. This set of factors makes treating these infections very difficult, demonstrating that developing new methods and compounds that are effectively antibacterial is essential.

Given the wide variety of molecules plants produce, many plant extracts have bactericidal or bacteriostatic potential, depending on their extraction method and plant organ [176]. Phenolic compounds, for example, are a set of essential substances found in natural plant extracts, and these are known to accumulate and destabilize bacterial cell walls [177]. In this context, there is a comprehensive and controversial discussion in the literature about the difference in susceptibility of Gram-negative and Gram-positive strains concerning the action of secondary plant metabolites, since different studies demonstrate that there is greater antimicrobial effectiveness of certain natural compounds of plants in distinct strains within this classification [178,179,180]. Furthermore, the presence of an outer membrane in Gram-negative strains and a thicker peptidoglycan wall, absent in the outer membrane in Gram-positive strains, makes the interactions between the natural compounds and the bacterial cell particular to each set of experimental parameters and strains used [177].

The cell membrane is a selectively permeable barrier, which, together with the cell wall, when present, is an important interaction interface between the microorganism and the molecules present in the extracts. In this way, many extracts will affect the membrane’s properties, leading to cell death. For example, ethanolic and water extracts of roselle (*Hibiscus sabdariffa*), rosemary (*Rosmarinus officinalis*), clove (*Syzygium aromaticum*), and thyme (*Thymus vulgaris*) reduced the cytoplasmic pH. They caused membrane hyperpolarization that could damage bacterial cell membranes [181]. Similarly, water garlic (*Allium sativum*) extracts caused membrane permeability and morphological changes in *Erwinia carotovora* bacteria [182]. Similar results were observed in the *Polygonum cuspidatum* [183], *Graptophyllum glandulosum* [184], and *Crataegus pinnatifda* [185] extracts. Protein inactivation and Reactive Oxygen Species are also important bactericidal mechanisms and were important to some extracts [186]. 

The use of a complex plant extract that has dozens of different compounds instead of a single isolated natural or even synthetic compound mimicking what is found in nature can bring about a decrease, addition, or synergy to the desired antimicrobial effect, and all this must be evaluated when analyzing the research of studies and developing these biomedical applications. Another important issue related to the mechanisms of action of these compounds is the mechanisms of antimicrobial resistance that can attenuate or prevent the effective action of natural extracts. For example, in addition to acting on bacterial cell walls, phenolic compounds, among other secondary metabolites, can chelate metals, fight free radicals, act as antioxidants, and, within bacterial cells, can inhibit the synthesis of nucleic acids, interact with proteins important for bacterial survival, proliferation, and dissemination, as well as impact bacterial quorum sensing and the biofilm formation of certain pathogenic species [187,188]. However, resistance mechanisms such as efflux pumps can prevent the effective action of phenolic compounds in strains rich in this molecular type of resistance by decreasing the necessary concentration of the compound in the cell interior when expelling the compound to the extracellular environment [177].

**Figure 2:**
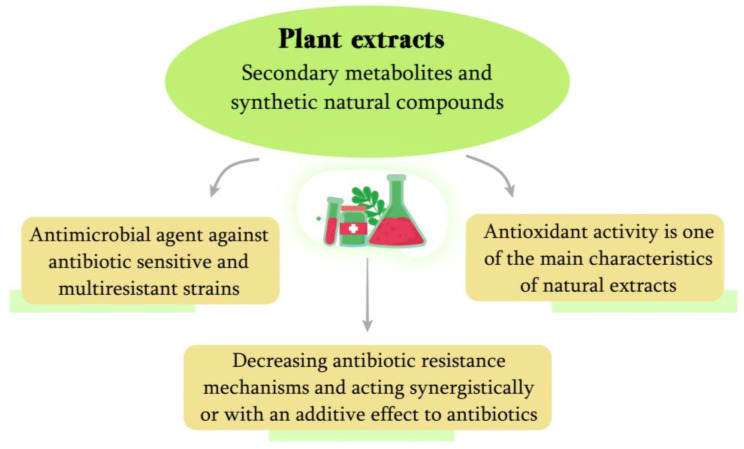
Biological activities of plant extracts.

From another point of view, phenolic compounds can also reduce or extinguish resistance mechanisms to antibiotics. Therefore, they can be used together with the traditional and most used antibiotics to increase their effectiveness or make them useful, at public health levels, for example, for a longer period of time. Antibiotic resistance mechanisms can be degradation or modification of the antibiotic, sequestration, and isolation of the antibiotic by the bacterial cell, and bacterial modification of the target on which the antibiotic acts, among others [189]. For example, phenolic compounds can combat the genus *Staphylococcus* and can decrease the minimum inhibitory concentration (MIC) of antibiotics against Methicillin-resistant *Staphylococcus aureus* (MRSA) strains [190]. In addition, it is noteworthy that multiresistant strains of antibiotics, even with different resistance mechanisms, may still be susceptible to the action of phytochemical compounds [191]. Other phenolic compounds, alone or together with multiple chemical species in plant extracts, can act synergistically with antibiotics of different classes and potentiate their action [192,193]. In this way, the antimicrobial action of plant extracts has several applications and potential when they are used alone as antibacterial agents or in synergy with traditional antibiotics already used in community and hospital infections, as shown in Figure 2.

Some different extractive methods and solvents can make the extract more or less antimicrobial due to the number of polyphenols and bioactive chemical species obtained. For example, the ethanolic extract of *Crataegus monogyna* leaf has antimicrobial activity against *Staphylococcus aureus*, having its MIC 0.512–1.024 mg/mL. However, extractions using this leaf with an aqueous solvent also showed antimicrobial activity for the *E. coli* species, which, on the other hand, with the ethanolic extract, did not demonstrate any activity [194]. Organic CHCl3/MeOH extracts from *H. glomerata* leaves showed antibacterial activity against *Staphylococcus aureus* and *Enterococcus faecium* and the resistant bacteria *K. pneumoniae* extended-spectrum β-lactamases (ESBL) [195]. 

The aqueous extract of the flower of *Acacia Saligna* (Labill.) H. L. Wendl., on the other hand, has antibacterial action against strains that cause diseases in plants and humans, such as *A. tumefaciens* and *E. cloacae*, being relevant for both applications. In addition to these examples, several other plants, using their different organs for extractions, have described antibacterial activities against sensitive and resistant strains to antimicrobials, demonstrating the technical–scientific relevance of the study and development of antimicrobial technologies from natural extracts and their bioactive compounds for medicine and other industries such as food and agriculture [196,197].

## 3. Plant-Based Antibacterial Green Nanomaterials

Nanotechnology is a multidisciplinary science that allows for the manipulating of matter on an atomic and molecular scale to develop new applications. This science impacts different sectors of society, ranging from agriculture to medicine. Furthermore, nanotechnology is expected to mobilize the economy, with more than 125 billion dollars in 2024, as this market is increasingly growing in various areas [23]. Among the vastness of products and applications of nanotechnology, nanomaterials have a clinical potential in medicine due to their nanoscale. This causes nanomaterials to present physicochemical characteristics, such as size, morphology, charge, luminescence, and magnetism, in addition to electronic and optical characteristics different from materials with larger scales [198]. Nanomaterials are a set of atoms bonded together, about 10–10^5^ atoms, with at least one dimension from 1 to 100 nm [199]. 

The synthesis of the nanomaterial is crucial for its cytotoxicity, physicochemical characteristics, and biological properties. Traditional syntheses of nanomaterials use compounds and solvents that are toxic and harmful to human and animal health, and the environment. The adversities surrounding traditional syntheses with toxic solvents involve problems from production to disposal, since all the toxic input produced will follow a course to the fauna, flora, oceans, and terrestrial layers [200]. However, the green synthesis of nanomaterials consists of organic compounds, such as plant extracts and solvents of zero or low toxicity, through an eco-friendly approach to the entire life cycle of the nanomaterial [201,202]. To carry out the synthesis, it is necessary to have a precursor source that can have an inorganic, organic, or metallic composition. Next to the precursor, it is necessary to have organic compounds, such as biomolecules, that are capable of reducing and stabilizing the ionic precursors to atoms. The first stage of green synthesis is called bioreduction. The bioactive (reducing agents) present in plant extracts, such as flavonoids, carotenoids, and polysaccharides, can reduce precursor ions to atoms. This step is fundamental for the biological property and cytotoxicity of the nanomaterial. Ions are more reactive than atoms due to the greater possibility of interaction. After bioreduction, the second step is nucleation. In this step, the atoms are joined together in synergy with the shielding of bioactivity, forming several nanoparticles. These nanoparticles form a nanosystem with bioactivity that stabilizes the synthesis process. During the preparation of the green synthesis, several physical–chemical factors (luminosity, thermal and electrical energy, pH, among others) can influence the stability of the nanoparticles and the properties that the nanomaterials will obtain [203,204,205,206,207,208,209]. 

**Figure 3:**
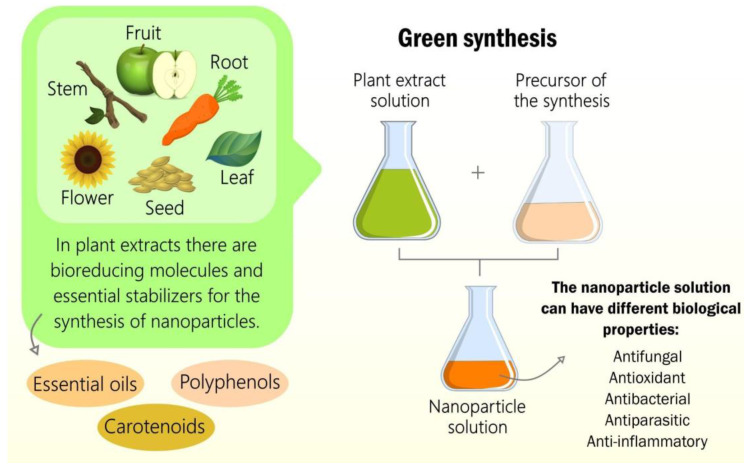
The green synthesis of nanomaterials from plant extracts with different biological properties.

In this sense, natural extracts are responsible for the reduction and stabilization of atoms through their biomolecules to form nanomaterials. These extracts can be of animal origin, for example, bee products, but they can also come from by-products produced by microorganisms and algae [210]. However, due to the dynamic and economical synthesis, the green synthesis of nanomaterials from plant extracts is used more frequently. These extracts can be made from a plant organ or synergy between several organs, such as leaves, fruits, seeds, roots, stems, and flowers. All these plant organs have different fundamental structures in the green synthesis process, such as essential oils, carotenoids, and phenolics responsible for the nanosystem’s antioxidant potential, as illustrated in Figure 3 [210].

It is noteworthy that seasonality, agrochemicals (fertilizers and pesticides), and environmental pollution are factors that can affect the plant extracts used to synthesize nanoparticles [209]. In environmental pollution, such as acid rain, the plant can suffer damage to the leaves that alters wettability, pubescence, and morphophysiological characteristics, such as reducing glandular trichomes [209,211]. Likewise, seasonal variations and climate changes can alter the size, availability, and development of leaves, flowers, fruits, and roots, among other plant organs. The secondary metabolism of plants is related to phenology, growing conditions, and seasonality. Therefore, the availability of bioactives may vary according to climate, light, temperature, soil nutrients, and agricultural treatments, such as agrochemicals [212,213,214,215]. Therefore, the characteristics of the plant can be altered by different factors, causing modification, loss, or excess of bioactive elements necessary for the green synthesis of nanoparticles. 

Many plants have efficient antimicrobial activities, depending on the bioactive extraction protocol. In this sense, the interaction with plant extracts and nanostructures can be an essential factor in activating potentialities associated with antibacterial agents. Furthermore, using only plant extracts has proven efficiency in the literature [177,191,216,217,218], but the synergistic effects between plant-based nanostructures by green synthesis have also been proven. As an example, we can mention the study of the green synthesis of gold nanoparticles (AuNPs) made from the extract (leaves and fruits) of *Pistacia atlantica*. AuNPs with a size between 40 nm and 60 nm and circular morphology showed antibacterial properties in Gram-negative bacteria *Escherichia coli* and *Pseudomonas aeruginosa* and Gram-positive bacteria *Staphylococcus aureus* and *Bacillus subtilis*. Notably, these are prevalent bacteria in hospital environments and are associated with cases of multidrug resistance. This nanosystem showed no cytotoxicity in Hela NCBI-C115 cells (Human cervix carcinoma) and obtained an antioxidant potential from assays that demonstrated the scavenging of free radicals such as 1,1-diphenyl-2-picryl-hydrazil (DPPH) [219,220]. The action of magnesium oxide nanoparticles (MgONPs) made from Saussurea costus root extract for different biological properties was also analyzed. The synthesis showed no cytotoxicity for mammalian cells, indicating that it is possible to synthesize biocompatible MgONPs. The nanoparticles, with cubic morphology and a size between 30 nm and 34 nm, showed antibacterial activity against *Escherichia coli, Pseudomonas aeruginosa, Bacillus subtilis*, and *Staphylococcus aureus*, in addition to fungal activity against strains *Candida tropicalis* and *Candida glabrata*. The same synthesis route exhibited anticancer activity against MCF-7 cancer cells [221]. In another study, bioactive compounds from the aqueous extract of *Medicago sativa* were investigated to synthesize nickel nanoparticles (NiNPs). It was observed that in bioactive compounds such as flavonoids, reducing sugars, proteins, and polysaccharides decreased after the green synthesis of NiNPs. This indicates that these bioactivities may be related to the bioreduction of nickel to NiNPs during green synthesis [222]. The same plant species has been used in ZnO nanoparticles (ZnONPs) and has been shown to have antimicrobial activity in strains of *Lactococcus lactis, Lactobacillus casei,* and *Staphylococcus epidermidis* and against the fungus *Candida albicans* [223]. Table 3 lists different plant-based nanomaterials by green synthesis. It is possible to highlight many essential and recent studies that act efficiently for Gram-positive and Gram-negative bacteria.

In order to better understand the functioning of nanoparticles, it is necessary to investigate the different mechanisms of antimicrobial action of nanomaterials. Among them, we can mention some caused by metallic nanoparticles, such as damage to proteins, inhibition of bacterial cell wall synthesis, disruption of the bacterial cell membrane, DNA damage, generation of reactive oxygen species, and prevention of bacterial biofilm formation, among other mechanisms [245]. 

Nanoparticles of various materials have different mechanisms of action through their synergistic or additive properties to natural extracts used as dispersive means and stabilizers of the nanosystem [246]. For example, NiO nanoparticles ranging in size from 2 to 16 nm were biosynthesized using *Stevia rebaudiana* leaf extract and demonstrated antibacterial activity against *Streptococcus mutans* [247]. On the other hand, the green synthesis of silver nanoparticles, with a size of 15 to 20 nm with a cubic crystalline structure, from the aqueous extract of *Coptis chinensis* rhizome and the modification of the surface of the nanomaterial with a chitosan biopolymer, led this antibacterial nanosystem against *E. coli* and *Bacillus subtilis* through cell wall damage and generation of reactive oxygen species, causing damage and release of biomolecules and bacterial intracellular structures [248]. The same effect can be observed in AgNPs biosynthesized by *Ocimum gratissimum* leaf extract against *E. coli* and *S. aureus* bacteria, having the same mechanisms of action [249]. Copper oxide nanoparticles (CuONPs) also have several green synthesis routes from plant extracts and diverse mechanisms of action, such as oxidative stress, protein, and genetic damage to bacteria [250]. As a last example, we can mention the ZnO nanoparticles synthesized using the extract of *Dysphania ambrosioides*, with a size of 5 to 30 nm and an almost spherical shape and hexagonal crystalline structure, and with antibacterial properties similar to the commercial ZnONPs used as a reference in the study. The *Prevotella intermedia* bacteria were the most sensitive to ZnONPs, with the most common mechanisms of action being the disruption of the cell wall and vital molecules of the cell, such as enzymes, DNA, and other proteins [251].

In this context, the synergistic effect between an antimicrobial plant extract and metallic nanoparticles synthesized in this dispersive medium potentiates the bactericidal and bacteriostatic activities of the technologies and studies developed, extending the synergistic effect with antibiotics, including against multi-antibiotic-resistant bacterial strains [252,253]. In this way, using nanotechnology synthesized from plant extracts presents itself as a potential alternative to the increasing occurrence of nosocomial infections multiresistant to antibiotics and as a key to combating bacterial dissemination through different technological applications made possible in several studies developed over the last few decades. However, it is noteworthy that studying the mechanisms of action from the interaction between nanosystems and bacteria still lacks more details for developing nanotechnologies addressed to mechanisms and actions in specific pathogenic strains.

## 4. Conclusions

Plant extracts and their therapeutic activities have been applied since the beginning of humanity through the traditional knowledge of their health benefits, even if, at the time, little knowledge existed to justify their use. Over time and with technological advances, scientists sought to understand the main compounds that offer plant extracts medicinal properties. Plant extracts and essential oils feature various molecules with a broad spectrum of activities not limited to pharmacological potential, but that can also be applied in the food industry, cosmetics, agriculture, and other fields.

Furthermore, these plant derivatives can be employed in the production of nanomaterials with a variety of applications. The synthesis using plant products has been widely investigated in recent years, demonstrating a variety of morphologies, sizes, and properties. As reported in the literature, green synthesis reduces the toxicity of the nanomaterial and the production of by-products throughout the entire process, and consumes less energy. However, an important point to be considered in this synthesis methodology is the type of extraction as well as the adopted preparation of plant extract and essential oil.

The type of extraction dramatically influences the type of bioactive compounds that will be isolated, consequently affecting its pharmacological properties and ability to create a suitable medium for the synthesis and stabilization of nanostructures. Therefore, it is essential to consider the physicochemical characteristics of the molecules of interest, such as solubility and stability at high temperatures. In this context, identifying such substances through advanced characterization techniques is essential. 

Once the intended molecules with specific characteristics are identified, it is possible to optimize the extraction protocols to increase the yield of the molecules of interest. However, it is important to emphasize that these plant products are chemically complex, containing a variety of molecules with lesser or greater abundance depending on the plant organ of origin and extraction method. This complexity can be fundamental to synthesizing nanomaterials effectively in some extracts, so that some molecules can act by reducing the metallic precursor while others play the role of stabilizer, preventing aggregation. Therefore, in some cases, minimizing this complexity may affect the synthesis efficiency of nanostructures in the plant extract or essential oil. In this way, nanotechnology based on green synthesis is of great interest in terms of the environment, variety of products, and applications that can be developed.

## Figures and Tables

**Table 1:** Extraction of polyphenols through different modern extraction techniques.

Extraction Techniques	Vegetal Materials	Solvent Extraction Ratio	Extraction Conditions	References
Liquid–liquid extraction	Olive mill wastewater	-	Ethyl acetate Temperature: 25 °C Extraction time: 20 min Four extraction cycles	[77]
Solid–liquid extraction	Olive pomace	Solvent-to-feed ratio: 1:5 (*w*/*v*) Ethanol, pH 2	Temperature: 25 °C Time: 180 min	[78]
UAE	Jussara pulp	Solvent-to-feed ratio: 20 mL/g 50% ethanol in distilled water pH 3	Time: 20 min Temperature: 25 °C	[63]
UAE	Grape skins	Solvent-to-feed ratio: 1:10 (*w*/*v*) Ethanol: water 50:50 *v*/*v*	Temperature: 25 °C Time: 9 min	[79]
UAE	Wine lees	Solvent-to-feed ratio: 1:60 (*w*/*v*) Ethanol 43.9%	Temperature: 60 °C Time: 25 min	[80]
MAE	Apple pomace	Solvent-to-feed ratio: 22.9:1 Ethanol concentration: 62.1%	Microwave power: 650.4 W Extraction time: 53.7 s Temperature: 70 °C	[81]
PLE	Grape pomace	-	Solvent extraction: ethanol: water 50:50 *v*/*v* Temperature: 80 °C Time: 50 min Pressure: 100 bar	[82]
EAE	Grape seeds	Enzyme dosage: 20 mg/g Water	pH 3.55 Lallzyme EX-V Enzyme dosage: 20 mg g^−1^ Temperature: 48 °C Time: 2.60 h	[83]
EAE	Corn husk	1 g plant to 10 mL of 0.1 M PCB at pH 5.0 Cellulase	Incubation: 40 °C Inactivation of the enzyme in boiling water for 5 min Solvent extraction: ethanol Time: 2 h Temperature: 80 °C	[84]
PEF	*Cistus creticus*	1 g plant in 10 mL solvent	PEF treatment time: 20 min Following extraction step: magnetic stirrer (500 rpm) and heated at 50 °C (in an oil bath) for 3 h	[50]

**Table 2:** HPLC experimental conditions for the determination of some classes of phenolic compounds.

Compounds	Stationary Phase	Column Temperature (°C)	Mobile Phase	Flow Rate of the Mobile Phase (mL/min)	Wavelength (nm)	References
Phenolic acids	C18 (4.6 mm × 150 mm, 5 μm)	25	(A) 0.1% formic acid (B) 100% methanol	0.7	270	[90]
Phenols	C18 (2.1 mm × 150 mm, 5 μm)	30	(A) H_2_O (0.1% *v*/*v* HFor) (B) MeOH 100% (0.1% *v*/*v* HAcO) Gradient elution	0.5	320, 280	[87]
Polyphenols Flavonoids Phenolic acids	C18 (4.6 mm × 150 mm, 5 μm)	-	(A) Methanol (B) Acetic acid:water (1:99, *v*/*v*)	1	210–400	[91]
Anthocyanins Polyphenols	Superspher 100 RP, (250 × 4.6 mm 18.5 μm)	30	(A) 10% formic acid in water (B) Methanol:water:formic acid (45:45:10, *v*/*v*/*v*)	0.8	530	[92]
Anthocyanins	C18	20	(A) Water:formic acid:ACN (87:10:3, *v*/*v*/*v*) (B) Water:formic acid:CAN (40:10:50, *v*/*v*/*v*)	1	520	[93]
Polyphenols	C18 (4.6 mm × 250 mm, 5 μm)	40	(A) water containing 0.5% *v*/*v* formic acid (B) acetonitrile:water (6:4) containing 0.5% *v*/*v* formic acid Gradient elution	1	220–360	[50]
Flavonols	Polar-RP 80A (150 mm × 4.6 mm 5 μm)	30	(A) water with 0.1% formic acid (B) methanol with 0.1% formic acid Gradient elution	0.8		[94]

**Table 3:** Examples of plant-based green synthesis of nanoparticles with antibacterial properties.

Nanomaterials	Plant-Based Green Synthesis of Nanoparticles	Antibacterial Properties	References
AgNPs	*Acacia lignin*	Antibacterial activity against *Escherichia coli, Pseudomonas aeruginosa, Bacillus subtilis Staphylococcus aureus, Bacillus circulam,* and *Ralstonia eutropha.*	[224]
AgNPs	*Dodonaea viscosa*	Antibacterial activity against *Streptococcus pyogene.*	[225]
AgNPs	*Euterpe oleracea*	Antibacterial dressings against *Staphylococcus aureus* and *Escherichia coli.*	[226]
AgNPs	*Pedalium murex*	Antibacterial activity against *Bacillus subtilis, Staphylococcus aureus, Escherichia coli, Micrococcus flavus, Pseudomonas aeruginosa, Klebsiella pheumoniae,* and *Bacillus pumilus.*	[227]
AgNPs	*Beta vulgaris*	Antibacterial textiles against *Staphylococcus aureus, Staphylococcus epidermidis,* and *Escherichia coli.*	[228]
AuNPs	*Pistacia atlantica*	Antibacterial activity against *Staphylococcus aureus, Bacillus subtilis, Escherichia coli,* and *Pseudomonas aeruginosa.*	[220]
AuNPs	*Amorphophallus paeoniifolius*	Antibacterial activity against *Escherichia coli, Citrobacter freundii, Bacillus subtilis, Pseudomonas aeruginosa, Salmonella typhimurium,* and *Staphylococcus aureus.*	[229]
AuNPs	*Jatropha integerrima* Jacq.	Antibacterial activity against *Bacillus subtilis, Staphylococcus aureus, Escherichia coli,* and *klebsiella pneumoniae.*	[230]
AuNPs	*Citrus maxima*	Antibacterial activity against *Staphylococcus aureus,* and *Escherichia coli.*	[231]
Al_2_O_3_NPs	*Prunus xyedonesis*	Antibacterial activity against *Pseudomonas aeruginosa.*	[232]
Al_2_O_3_NPs	*Cymbopogan citratus*	Antibacterial activity against *Pseudomonas aeruginosa.*	[233]
MgONPs	*Sargassum wightii*	Antibacterial activity against *Streptococcus pneumonia, Escherichia coli, Pseudomonas aeruginosa,* and *Aeromonas baumannii.*	[234]
MgONPs	*Annona squamosa*	Antibacterial activity against *Pactobacterium carotovorume.*	[235]
MgONPs	*Rhododendron arboreum*	Antibacterial activity against *Escherichia coli, Spectrococous mutans,* and *Proteus vulgaris.*	[236]
MgONPs	*Saussurea costus*	Antibacterial activity against *Staphylococcus aureus, Bacillus subtilis, Escherichia coli*, and *Pseudomonas aeruginosa.*	[221]
ZnONPs	*Pongamia pinnata*	Antibacterial activity against *Pseudomonas aeruginosa.*	[237]
ZnONPs	*Ailanthus altissima*	Antibacterial activity against *Staphylococcus aureus* and *Escherichia coli.*	[238]
ZnONPs	*Medicago sativa* L.	Antibacterial activity against *Staphylococcus epidermidis, Lactococcus lactis,* and *Lactobacillus casei.*	[223]
TiO_2_NPs	*Psidium guajava*	Antibacterial activity against *Staphylococcus aureus* and *Escherichia coli.*	[239]
TiO_2_NPs	*Mentha arvensis*	Antibacterial activity against *Escherichia coli, Proteus vulgaris,* and *Staphylococcus aureus.*	[240]
TiO_2_NPs	*Trigonella foenum-graecum*	Antibacterial activity against *Staphylococcus aureus*, *Enterococcus faecalis*, *Klebsiella pneumoniae, Streptococcus faecalis, Pseudomonas aeruginosa, Escherichia coli, Proteus vulgaris, Bacillus subtilis,* and *Yersinia enterocolitica.*	[241]
TiO_2_NPs	*Azadirachta indica*	Antibacterial activity against *Salmonella typhi* and *Escherichia coli.*	[242]
TiO_2_NPs	*Hypsizygus ulmarius*	Antibacterial activity against *Escherichia coli, Staphylococcus aureus*, *klebsiella pneumoniae,* and *Bacillus cereus.*	[243]
TiO_2_NPs	Pristine pomegranate peel extract	Bacterial disinfection against *Escherichia coli, Staphylococcus aureus*, and *Pseudomonas aeruginosa.*	[244]

## Data Availability

Not applicable.
